# Longitudinally Tracking Maternal Autonomic Modulation During Normal Pregnancy With Comprehensive Heart Rate Variability Analyses

**DOI:** 10.3389/fphys.2022.874684

**Published:** 2022-05-09

**Authors:** Maretha Bester, Rohan Joshi, Massimo Mischi, Judith O. E. H. van Laar, Rik Vullings

**Affiliations:** ^1^ Department of Electrical Engineering, Eindhoven University of Technology, Eindhoven, Netherlands; ^2^ Patient Care and Monitoring, Philips Research, Eindhoven, Netherlands; ^3^ Department of Obstetrics and Gynecology, Máxima Medical Centrum, Veldhoven, Netherlands

**Keywords:** obstetric and gynecologic, heart rate vaiability, autonomic nervous system, maternal health, pregnancy

## Abstract

Changes in the maternal autonomic nervous system are essential in facilitating the physiological changes that pregnancy necessitates. Insufficient autonomic adaptation is linked to complications such as hypertensive diseases of pregnancy. Consequently, tracking autonomic modulation during progressing pregnancy could allow for the early detection of emerging deteriorations in maternal health. Autonomic modulation can be longitudinally and unobtrusively monitored by assessing heart rate variability (HRV). Yet, changes in maternal HRV (mHRV) throughout pregnancy remain poorly understood. In previous studies, mHRV is typically assessed only once per trimester with standard HRV features. However, since gestational changes are complex and dynamic, assessing mHRV comprehensively and more frequently may better showcase the changing autonomic modulation over pregnancy. Subsequently, we longitudinally (median sessions = 8) assess mHRV in 29 healthy pregnancies with features that assess sympathetic and parasympathetic activity, as well as heart rate (HR) complexity, HR responsiveness and HR fragmentation. We find that vagal activity, HR complexity, HR responsiveness, and HR fragmentation significantly decrease. Their associated effect sizes are small, suggesting that the increasing demands of advancing gestation are well tolerated. Furthermore, we find a notable change in autonomic activity during the transition from the second to third trimester, highlighting the dynamic nature of changes in pregnancy. Lastly, while we saw the expected rise in mean HR with gestational age, we also observed increased autonomic deceleration activity, seemingly to counter this rising mean HR. These results are an important step towards gaining insights into gestational physiology as well as tracking maternal health via mHRV.

## 1 Introduction

The period of pregnancy necessitates major physiological changes to sustain the growing fetus while maintaining maternal health (Khlybova et al., 2008; Moors et al., 2020). Some changes are apparent and can be readily monitored, such as the mother’s growing abdomen. Abdominal measurements (i.e., symphysial fundal height) are typically taken at prenatal checkups to track the progressing pregnancy and the growing fetus (Yousif et al., 2019). Other changes are internal, such as the substantial adaptations in the maternal cardiovascular system, which are largely regulated by the autonomic nervous system (ANS) (Khlybova et al., 2008; Cui et al., 2018; Shanmugalingam et al., 2019). Similar to tracking the symphysial fundal height, longitudinal assessment of autonomic modulation throughout pregnancy may offer insights into gestational health (Khan et al., 2006).

In fact, several pregnancy complications are linked to the insufficient adaptation of the ANS to advancing gestation. Complications such as preeclampsia (a hypertensive disorder of pregnancy) and preterm birth have been associated with dysfunctional maternal autonomic regulation (Khlybova et al., 2008; Yousif et al., 2019; Moors et al., 2020). These complications are challenging to detect in early gestation when available interventional options (such as aspirin for mitigating preeclampsia) would be most effective (Cui et al., 2018; Shanmugalingam et al., 2019). As a result, such pregnancy complications remain major causes of perinatal morbidity and mortality (Khan et al., 2006; Saleem et al., 2014; Frey and Klebanoff, 2016; Umesawa and Kobashi, 2017).

Alleviating the burden of pregnancy complications partly depends on developing screening methods for their early detection (Kleinrouweler et al., 2016; De Kat et al., 2019). Owing to the association between pregnancy complications and autonomic dysfunction, tracking autonomic changes throughout pregnancy may allow for detecting deteriorations in maternal health (Antonazzo et al., 2004; Pal et al., 2009). However, the normative values of autonomic activity and the trajectory thereof during pregnancy remains insufficiently explored (Brooks et al., 2020; Garg et al., 2020).

Autonomic activity can be assessed by tracking heart rate variability (HRV) since autonomic regulation modulates the time-intervals between heartbeats (Task Force of The Europea, 1996; Shaffer and Ginsberg, 2017). Tracking HRV is attractive due the pervasiveness of unobtrusive, wearable technologies that can monitor heart rate (HR) (Grym et al., 2019). However, limited research has longitudinally assessed maternal HRV (mHRV) in healthy pregnancy (Schlembach et al., 2012). Moreover, existing research has offered conflicting results (Yousif et al., 2019; Garg et al., 2020; Moors et al., 2020). On the one hand, some researchers have found that mHRV – and by proxy, autonomic activity—is unaffected by gestational age (GA) (Voss et al., 2000; Moertl et al., 2009). On the other hand, the findings of other researchers suggest an increased activity of the parasympathetic branch of the ANS in early gestation with a shift towards sympathetic dominance by the end of pregnancy (Balajewicz-Nowak et al., 2016; Kolovetsiou-Kreiner et al., 2018; Garg et al., 2020).

Changes in maternal physiology during pregnancy are complex and dynamic (Brooks et al., 2020). Consequently, regular prenatal checkups, initially monthly and culminating in weekly appointments, are necessary to capture possible changes (American Academy of Pedia, 2017). Although measuring the abdomen’s symphysial fundal height during these checkups provides valuable information on the health of the pregnancy, it is not the only information considered. Maternal blood pressure, fetal HR and fetal growth are also assessed to generate a more comprehensive overview of gestational health (Basis Prenatale Zor, 2015).

Similarly, assessing mHRV with more regularity by employing multiple measures of HRV may better showcase the progression of autonomic modulation in normal pregnancy. In literature, mHRV is typically assessed only three times (i.e., once per trimester) with a methodological focus on standard time and frequency domain features (Schlembach et al., 2012). These features inform on the relative activity of the sympathetic and parasympathetic branches of the ANS, which has been the focus of mHRV research in pregnancy. However, further information can be obtained from HRV. The variability observed in HR results from the interaction of a network of non-linear physiological systems over different time scales (Shaffer et al., 2014; Bakhchina et al., 2018). Calculating HRV features that exploit characteristics of these interactions—such as complexity, responsiveness, and fragmentation—may allow for a more comprehensive overview of maternal autonomic modulation during pregnancy (Silva et al., 2017).

While features that capture these characteristics have rarely been employed in assessing mHRV, they have been used elsewhere. For instance, sample entropy and detrended fluctuation analysis, which assess the complexity in the HR time series, have been used in research on sepsis, heart failure, sleep staging, and stress (Peng et al., 1995; Ahmad et al., 2009; Vigo et al., 2010; Bakhchina et al., 2018). Diseased states and stress typically result in reduced HR complexity. Pregnancy is often described as a stress-test for the mother owing to the increasing physiological strain accompanying advancing gestation (Williams, 2003; Bilhartz et al., 2011; Chasan-Taber, 2016). Consequently, measures of HR complexity may be particularly sensitive to progressing pregnancy.

Furthermore, the increased stress of pregnancy might affect the responsiveness of the ANS. Autonomic responsivity can be probed with phase rectified signal averaging (PRSA) (Bauer et al., 2006a), a method that quantifies how the tachogram responds to accelerations and deceleration in HR as a proxy measure for autonomic responsiveness. PRSA-based features not only independently predict mortality in cardiac disease (Bauer et al., 2006b; Eick et al., 2015) but are also sensitive to aging and fitness levels (Campana et al., 2010). This method is increasingly being employed to assess fetal health (Graatsma et al., 2012; Georgieva et al., 2014) but has rarely been used to assess maternal autonomic modulation (Tejera et al., 2012; Casati et al., 2016).

Lastly, physiologically stressful conditions (such as aging or cardiovascular disease) are associated with a breakdown in the neuroautonomic-electrophysiological control systems that regulate HR, resulting in increased short-term HRV (Costa et al., 2017). This high short-term variability can be misleading, since it indicates healthy autonomic modulation, which is not typically present in aging populations or those with cardiac disease. However, in cases of such breakdown the variation is fragmented—i.e., with HR quickly alternating between acceleration and deceleration—rather than gradual, as is inherent to vagally regulated variation. A recent class of HRV features, namely heart rate fragmentation (HRF), exploits this phenomenon to probe the integrity of the physiological systems controlling the heartbeats. HRF features outperform traditional HRV features in capturing the degenerative impact of conditions such as aging and heart disease (Costa et al., 2017). Assessing these features in pregnancy for the first time could indicate whether advancing gestation affects the physiological systems that control the heartbeat.

Subsequently, in this study, we will implement a variety of HRV methods and apply these repeatedly in a healthy pregnant population to investigate the progression of maternal autonomic modulation under the stress-test of advancing gestation. This investigation will not only strengthen our understanding of gestational physiology but also serve towards providing evidence for the trajectory of mHRV during healthy pregnancy that may, in turn, be used as a guideline for obstetric screening.

## 2 Methods and Materials

This research is a secondary analysis of a prospective observational study carried out from 2007 to 2009. Healthy women (18 years and older) with uneventful, singleton pregnancies were recruited before 12 weeks of gestation for participation (*n* = 40). Pregnancy duration was determined from the last menstrual period and then confirmed at 10–12 weeks of gestation by the crown-rump length. Participants took no medication apart from iron supplements or vitamins (van Laar et al., 2014). Women who developed pregnancy complications during the study were excluded from the final analysis (hypertension, *n* = 4; preterm birth, *n* = 3). Four participants were further excluded due to dropout from the study of datafiles that were missing. The data from the remaining 29 participants were included. These participants had a mean age of 31 (±4) years and a mean pre-pregnancy body mass index of 23.9 (±4) kg/m^2^, as seen in Table 1.

**TABLE 1:** Patient characteristics.

Characteristics	% Or Mean (Standard Deviation)
Maternal body mass index before pregnancy	23.9 (4) kg/m2
Nulliparous	66%
Maternal age at birth	31 (4) years
Gestational age at birth	40 weeks (10 days)

All participants provided written informed consent. The institutional review board at the Máxima Medical Center, Veldhoven, the Netherlands, approved the study (reference number 0650) and granted a waiver for this secondary analysis in 2021 (reference number N21.008). The study design and original analyses are detailed in a previous article (van Laar et al., 2014).

### 2.1 Data Acquisition

Fetal ECG measurements—which also capture maternal ECG—were acquired at 1000 Hz from the maternal abdomen with a non-invasive electrophysiologic monitor (the NEMO device, Maastricht Instruments, Netherlands) (van Laar et al., 2014). Repeated measurements were performed at approximately 14, 18, 22, 24, 26, 30, 34, 36, 38, and 40 weeks of gestation while the participant was lying in a semi-recumbent position. 45-min long measurements were performed between 08:00 and 18:00 h. Included participants had a median of eight measurement sessions (IQR: 7–9). Relevant patient metadata was also collected (van Laar et al., 2014).

### 2.2 Preprocessing

A 4th-order Butterworth bandpass filter (1–70 Hz) and a notch filter (50 Hz) were applied to the ECG recordings, as proposed in a previous publication (Rooijakkers et al., 2014). Next, maternal ECG data were isolated from fetal recordings by applying a fixed linear combination to enhance the maternal QRS complexes (Rooijakkers et al., 2014). Thereafter, a peak detection algorithm was employed as detailed by Rooijakkers et al. (2011) to determine the RR series, or tachogram. As this algorithm was originally designed for fetal ECG, relevant parameters were adapted as appropriate for maternal ECG by the original authors of the algorithm: the relative characteristic frequency of the wavelet was set to 19 and the HR limits to 30 and 210 beats per minute. Processing of maternal RR intervals from fetal ECG measurements was done in MATLAB (MathWorks, United States), with all further processing done in Python (PSF, United States). To further eliminate possible ectopic beats or motion artifacts and improve the accuracy of HRV features, RR intervals which fell outside a specified range (0.4–2 s) or differed from the preceding interval by 20% were removed (Peters et al., 2008; Campana et al., 2010; Peters et al., 2011). RR intervals for which both preceding and following value were excluded based on above criteria, were also excluded. In cases where more than 25% of RR intervals needed to be removed, the measurement was excluded from the analysis. For HRV features where beat-to-beat changes were highly important (i.e., time-domain features, PRSA, HRF and Poincaré analysis), beats were replaced with NaN values. Where signal continuity was of higher importance (i.e., sample entropy, detrended fluctuation analysis, and frequency-domain analyses), missing values were linearly interpolated.

### 2.3 Heart Rate Variability

A range of HRV features were calculated on the entire RR series for each measurement session: standard time and frequency domain features (Task Force of The Europea, 1996; Shaffer and Ginsberg, 2017), non-linear (i.e., geometrical and complexity) features (Peng et al., 1995; Richman and Moorman, 2000; Khandoker et al., 2013; Shaffer and Ginsberg, 2017), phase rectified signal averaging (PRSA) (Bauer et al., 2006a), and heart rate fragmentation (HRF) (Costa et al., 2017). The standard features and complexity features were calculated using pyHRV (Gomes et al., 2019), a Python signal processing toolbox shown to be reliable (Shaqiri and Gusev, 2020). For the frequency domain analysis, each RR series was divided into shorter, overlapping segments (5 min in length, 50% overlap) during computation, where it was assumed that during these shorter segments the RR series is stationary. The mean of the values computed per segment was taken as the result for the corresponding RR series. All HRV features, further detailed in the following sections, were calculated for each measurement session.

#### 2.3.1 Standard Time and Frequency Domain Features

• SDNN: standard deviation of all RR intervals
• RMSSD: root mean squared successive differences of RR intervals
• pNN50: percentage of pairs of consecutive RR intervals differing by more than 50 ms
• LF: the power in the low frequency band (0.04–0.15 Hz)
• HF: the power in the high frequency band (0.15–0.40 Hz)
• TP: the total power in the frequency bands
• LF/HF: the ratio between low and high frequency power

While SDNN represents overall variability, other features inform on the relative contributions of the sympathetic and parasympathetic branches of the ANS. RMSSD, pNN50 and HF reflect the vagal modulation of HR. Both sympathetic and parasympathetic activity contribute to LF while LF/HF is typically attributed to sympathovagal balance (Task Force of The Europea, 1996; Shaffer and Ginsberg, 2017).

#### 2.3.2 Non-Linear Features

In addition to standard time- and frequency-domain features, we evaluated methods designed to better capture the non-stationary and non-linear characteristics of the HR time series. We employed a Poincaré plot analysis, which is a popular geometrical method to evaluate HRV dynamics. Each NN interval is plotted against its predecessor, resulting in a scatter plot. SD1 denotes the standard deviation of the short-term NN interval variability. Similarly, SD2 represents the standard deviation of the long-term NN interval variability. The ratio between these two features is noted as SD1/SD2 (Khandoker et al., 2013).

Furthermore, we employ two measures that address the complexity within the HR time series: sample entropy (SampEn) and detrended fluctuation analysis (DFA) (Peng et al., 1995; Richman and Moorman, 2000). The first, SampEn, calculates the conditional probability that two epochs which are similar within a tolerance *r* for a window length *m*, will remain similar if the next data point (i.e., the next NN value) is included (Richman and Moorman, 2000; Bakhchina et al., 2018). It can be defined as follows:

$$SampEn=-\text{l}\text{o}\text{g}\frac{A}{B},$$ (1)

*A* is defined as the number of pairs of vector (*x*) for *m* points which satisfy the condition d[*xm*(i), *xm*(j)] ≤ *r*, while *B* is the number of pairs of vectors for (*m*+1) points that satisfies the same condition (Bakhchina et al., 2018). The values for *m* and *r* were set to 2 and 0.2 times the standard deviation of the RR intervals, as is typically reported in the literature (Richman and Moorman, 2000). Smaller values of SampEn indicate more regular and predictable time series (Shaffer and Ginsberg, 2017).

The second method, DFA, also gives an estimate of the long-range correlations of the time series by quantifying its fractal scaling properties follows (Peng et al., 1995). In short, the total time series is integrated ( $X_{t})$ and then divided into segments of length *n*. Each segment is then detrended by subtracting the best linear fit ( $Y_{t})$ . The fluctuation function is calculated as shown in Eq. 2.

$$F\left(n\right)=\sqrt{\frac{1}{N}}\sum_{\mathrm{t=}1}^{N}{\left(X_{t}-Y_{t}\right)}^{2},$$ (2)

Thereafter, the scaling exponent *α* (which represent the correlation properties of the time series) is estimated from the log-log plot of F(n) vs. *n*. Typically, both *α₁*, and *α₂* are determined, which represent short-term and long-term fractal scaling exponents. In our case, only *α₁* (which calculates correlation over *n* = 4–16 beats) is calculated, as *α₂* requires several hours of data to achieve sufficient accuracy. When there is no correlation present (i.e., white noise) or the signal resembles a random walk process (i.e., Brownian noise), *α* = 0.5 and *α* = 1.5, respectively. Positive correlations exist when 0.5 < *α* < 1.5, with *α* ≈ 1 suggesting a high level of complexity. When values start exceeding 1, it suggests that the system becomes increasingly regular (Peng et al., 1995; Yeh et al., 2009).

#### 2.3.3 Phase Rectified Signal Averaging

PRSA is a technique that can identify and elucidate quasi-periodicities in time-series data that are often obscured by noise and non-stationarities, as is typical for physiological signals (Bauer et al., 2006a). In PRSA, signal segments are aligned corresponding to a predetermined shared phase and then averaged, cancelling out the noise and isolating the underlying composite trend. These isolated quasi-periodicities are representative of the underlying physiological processes that regulate HR.

Isolating these quasi-periodicities is achieved in a few steps. Firstly, anchor points (APs) are defined on the RR series in relation to the phases that are of interest. Here, two sets of APs are identified, namely HR accelerations and decelerations. If the PRSA parameter of *T* is set to one, each acceleration and deceleration are marked as an AP. A higher value of *T* evokes a low pass filtering effect since then an AP would be identified as an acceleration or deceleration averaged over *T* points.

After the APs have been identified, a signal segment is defined around each AP with a length of *L* both preceding and following the AP (resulting in a total segment length of 2*L* + 1). This signal segment should be sufficiently long to capture the slowest anticipated oscillation of relevance in the time-series. We define *L* as 50 RR values, as is also done in the literature (Joshi et al., 2019). All signal segments are then aligned corresponding to their APs and averaged across segments, resulting in the PRSA waveform. This waveform (also of length 2*L* + 1 and consisting of averaged RR values) visualizes the behavior of HR in response to accelerations and decelerations, which is associated with sympathetic and vagal activity, respectively (Bauer et al., 2006b). In essence, the magnitude and speed of this response in HR gives an estimate of the robustness of the autonomic response (Bauer et al., 2006a).

To quantify this response, several features are calculated from the PRSA waveform, represented as X . Note that the PRSA waveform’s relationship to the time domain is units of RR values (specified here as RR_i_) and not in seconds. Firstly, deceleration capacity (DC) and acceleration (AC) are calculated to capture the magnitude of the response. The calculation of DC is shown in Eq. 3. AC is similarly calculated.

DC=[X(0)+ X(1)- X(-1)- X(-2)]/4, (3)

Here  X(0) represents the AP, X(1) is value following the AP, and X(−1) and X(−2) precede the AP (Bauer et al., 2006a). AC is similarly calculated. Furthermore, the immediate deceleration response (IDR) and immediate acceleration response (IAR) are calculated as the difference between the maximum and minimum RR_i_ within the neighborhood of five RR_i_ preceding the AP and five after, including the AP. This captures the maximum response in HR in the immediate neighborhood of the AP. The rate of this maximum response is captured in the slope of the deceleration and acceleration responses (SDR and SAR), which notes the slope of the line joining the maximum and minimum RR_i_ corresponding to IDR and IAR. Lastly, the average HR response to accelerations (AAR) and deceleration (ADR) is calculated by taking the difference between the mean of the 50 RR_i_ following the AP, which is included, and the mean the 50 preceding RR_i_ (Joshi et al., 2019)_._

Lastly, the PRSA waveform is also studied in the frequency domain. The power spectral density (PSD) plots are calculated for all PRSA waveforms, with the frequency content measured in time units of 1/RRi. Calculating the PSD of these rectified waveforms have been shown to perform better than traditional spectral analysis (Bauer et al., 2006a; Bauer et al., 2006b; Joshi et al., 2019). PRSA allows for separating the acceleration and deceleration response; subsequently, we calculate features to capture the ratio between the two responses to better understand autonomic control of the maternal HR. To this end, we firstly determine the power and peaks in the LF and HF frequency bands in the PSDs, as well as the TP for both the acceleration and deceleration response. Thereafter, we determine the ratio between the features (for example, HF_acc_:HF_dec_).

#### 2.3.4 Heart Rate Fragmentation

HRF aims to capture variability resulting from a breakdown in physiological control over HR rather than healthy autonomic modulation. Costa et al. (2017) first developed these indices to address the phenomenon of increased HR variability in older populations and populations with heart disease where vagal modulation is known to be decreased. Closer investigation revealed the variability to be jagged rather than smooth as would be expected from vagal control. Subsequently, a set of indices were developed to capture this jagged variation, referred to as fragmentation. These indices are: PIP (percentage inflection points); PAS (percentage alternating segments); PSS (percentage short segments); and IALS (inverse of accelerating or decelerating long segments). Increases in these indices indicate increased fragmentation in HR (Costa et al., 2017).

### 2.4 Data Analysis

Data were analyzed with two aims in mind. Firstly, we aimed to explore the possibly dynamic relationship between the HRV features and GA. To this end, we grouped HRV features into bins of 4 weeks. The mean and standard error of the mean of the HRV features for all participants per bin is plotted against GA to show the evolution of the features over time. The mean and standard error of the mean are preferred over the median and interquartile range since we are interested in the trajectory of the features and the support in each bin (i.e., the number of measurements) varies. However, due to the possibly non-normal distribution of the data, we also plot the median and interquartile range to confirm the trends observed. The data are grouped into the following GA bins, with the lower limit excluded and upper limit included: 12–16 weeks (19 measurements); 16–20 weeks (24 measurements); 20–24 weeks (31 measurements); 24–28 weeks (46 measurements); 28–32 weeks (28 measurements); 32–36 weeks (30 measurements); and above 36 weeks (52 measurements).

Secondly, we aimed to capture the significance and magnitude of changes observed. To this end, the data were divided into three groups based to facilitate comparison: less than 23 weeks (GA₁), between 23 and 32 weeks (GA₂), and over 32 weeks of gestation (GA₃). The groups span a comparative number of weeks and allow most participants to have at least one measurement per group. The upper limit is included and the lower excluded in each group. Participants typically had multiple measurements in each group; subsequently, the mean of these values was taken per participant per gestational group. One participant did not have a measurement in each of the three gestational groups and was subsequently excluded from this second part of the analysis, resulting in total of 28 participants.

### 2.5 Statistical Analysis

Physiological data is typically not normally distributed and therefore non-parametric analyses were done. We also confirmed the nature of the distribution by using a Kolmogorov-Smirnov test (only three out of 28 features were normally distributed). Subsequently, a Friedman’s test with a Dunn’s post hoc test was applied to determine whether statistically significant differences occurred across the three GA groups (i.e., GA₁, GA₂, and GA₃) as well as between individual groups (e.g., GA₁ vs. GA₂), with Bonferroni correction to control for family-wise error. This analysis test whether A value of *p* < 0.05 was considered statistically significant.

Corresponding effect sizes were calculated with Cohen’s U_1_, which provides a measure of the overlap between the distributions of two groups. A Cohen’s U_1_ of 1 indicates two entirely separate groups, while complete overlap results in a U_1_ of 0. While the standards for what constitutes a large effect are more clearly defined for Cohen’s d (used in parametric data), this is not the case for Cohen’s *U*_*1*_. A Cohen’s *d* of 0.2 (small effect) is similar to *U*_*1*_ = 0.15, while a d = 0.5 (medium effect) corresponds to *U*_*1*_ = 0.33 (Fritz et al., 2012).

## 3 Results

### 3.1 Time and Frequency Domain Features

**FIGURE 1:**
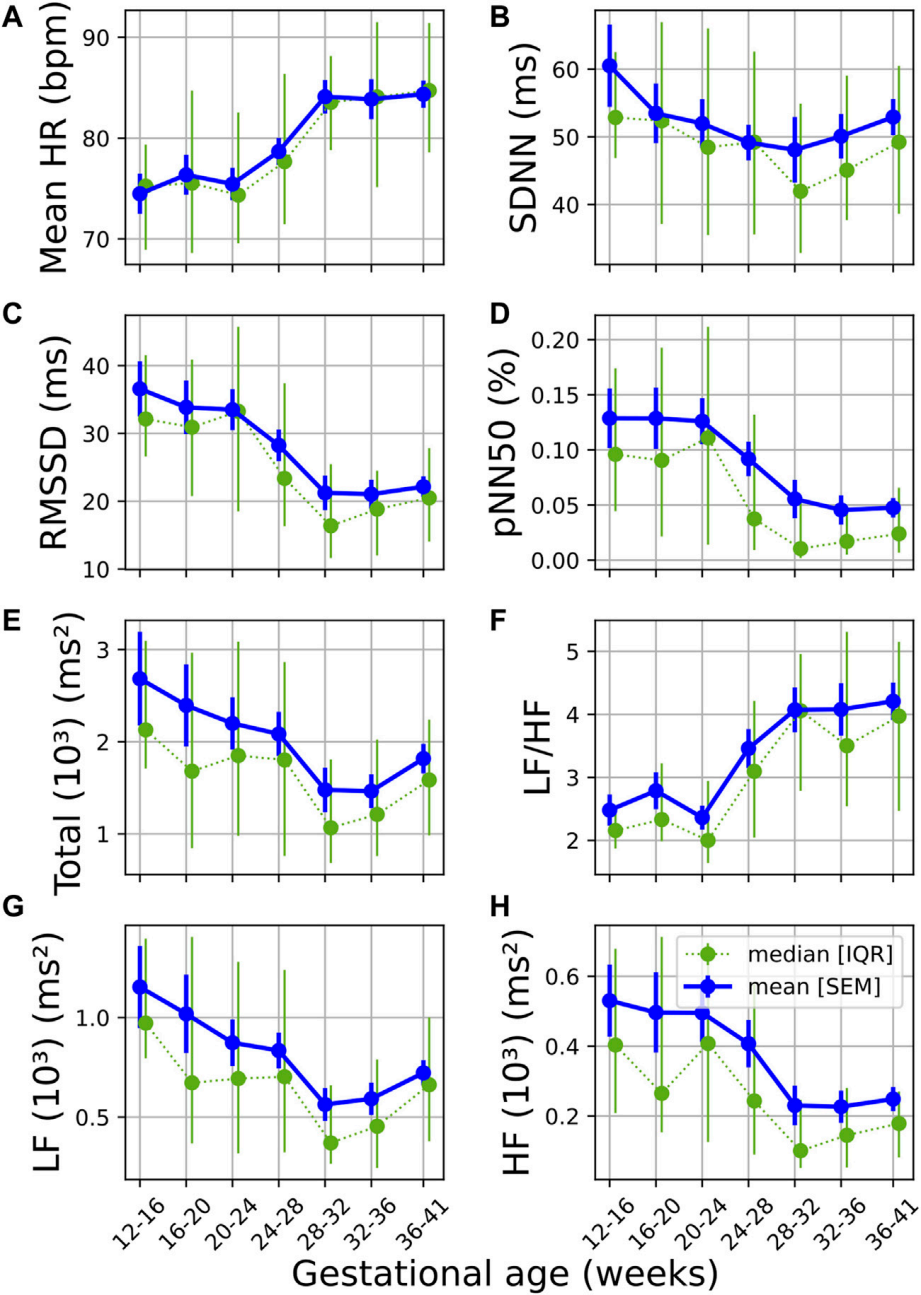
Temporal evolution of standard time and frequency domain measures over GA bins. HRV features for all participants were grouped into bins of 4 weeks. The mean and standard error of the mean (full line) as well as the median and interquartile range (dotted line) of the HRV features per bin is plotted against GA. **(A)** Mean HR; **(B)** SDNN; **(C)** RMSSD; **(D)** pNN50; **(E)** Total power; **(F)** LF/HF; **(G)** LF power; and **(H)** HF power.

Similar trends can be observed (Figure 1) across the temporal evolution of most standard time and frequency domain features. Mean HR and LF/HF increased significantly over pregnancy, RMSSD, pNN50, TP, LF and HF showed significant decreases with GA (Figure 1; Table 2). The change in pNN50 from GA₁ and GA₃ had the largest effect size (*U₁* = 0.196, Table 2), although this remains a small effect. The trend in overall variability (SDNN) was less distinct, showing first a decrease and thereafter an increase in values (Figure 1B; Table 2). Interestingly, the most notable changes in most features occur approximately between 24 and 32 weeks of gestation (Figure 1). All features except SDNN show sharp increases or decreases during this period.

**TABLE 2:** Results from the grouped analysis for standard time- and frequency-domain features.

Features	GA_1_ median (IQR)	GA_2_ median (IQR)	GA_3_ median (IQR)	Friedman *p*-value	GA_1_→GA_2_		GA_2_→GA_3_		GA_1_→GA_3_	
					*p*	*U*_1_	*p*	*U*_1_	*p*	*U*_1_
GA (weeks)	18.4 (18.2–20.3)	27.1 (26.7–27.7)	36.8 (36.1–37.4)							
HR (beats per minute)	74.6 (68.2–81.7)	79.8 (76.1–87.1)	85.5 (77.8–90.8)	**<0.0001**	0.056	0.161	0.577	0.036	**<0.001**	0.196
SDNN (ms)	53.2 (39.3–65.7)	47.0 (34.6–62.0)	48.6 (40.4–60.8)	**0.039**	0.750	0.054	1	0	1	0.054
RMSSD (ms)	32.9 (21.7–44.8)	25.4 (15.7–33.1)	20.7 (15.4–26.2)	**<0.0001**	0.103	0.125	1	0	**0.006**	0.125
pNN50 (%)	0.12 (0.03–0.20)	0.05 (0.01–0.12)	0.03 (0.01–0.06)	**<0.0001**	0.110	0.161	0.834	0	**0.004**	0.196
Total power (ms^2^)	2080 (1302–3220)	1632 (732–2484)	1557 (876–2188)	**<0.001**	0.533	0.054	1	0.034	0.322	0.072
LF (ms^2^)	738 (568–1376)	650 (304–1119)	635 (349–920)	**<0.001**	0.417	0.034	1	0.018	0.253	0.071
HF (ms^2^)	398 (193–757)	195 (98–433)	168 (83–289)	**<0.0001**	0.128	0.089	1	0.018	**0.019**	0.125
LF/HF	2.29 (1.77–2.89)	3.32 (2.30–3.91)	4.05 (2.7–5.38)	**<0.0001**	0.064	0.071	0.566	0.018	**<0.001**	0.107

All continuous data are presented as median (IQR). First, the Friedman’s *p*-value is calculated to determine whether significant changes occur over all groups. Thereafter, the Dunn’s post-hoc test with Bonferroni correction is applied to determine the *p*-value between groups. Cohen’s *U*_*1*_ is calculated to determine effect size. Values of approximately 0.15 and 0.33 represent small and medium effects, respectively.

The bold type indicates that they values are statistically significant.

### 3.2 Non-Linear Features

**FIGURE 2:**
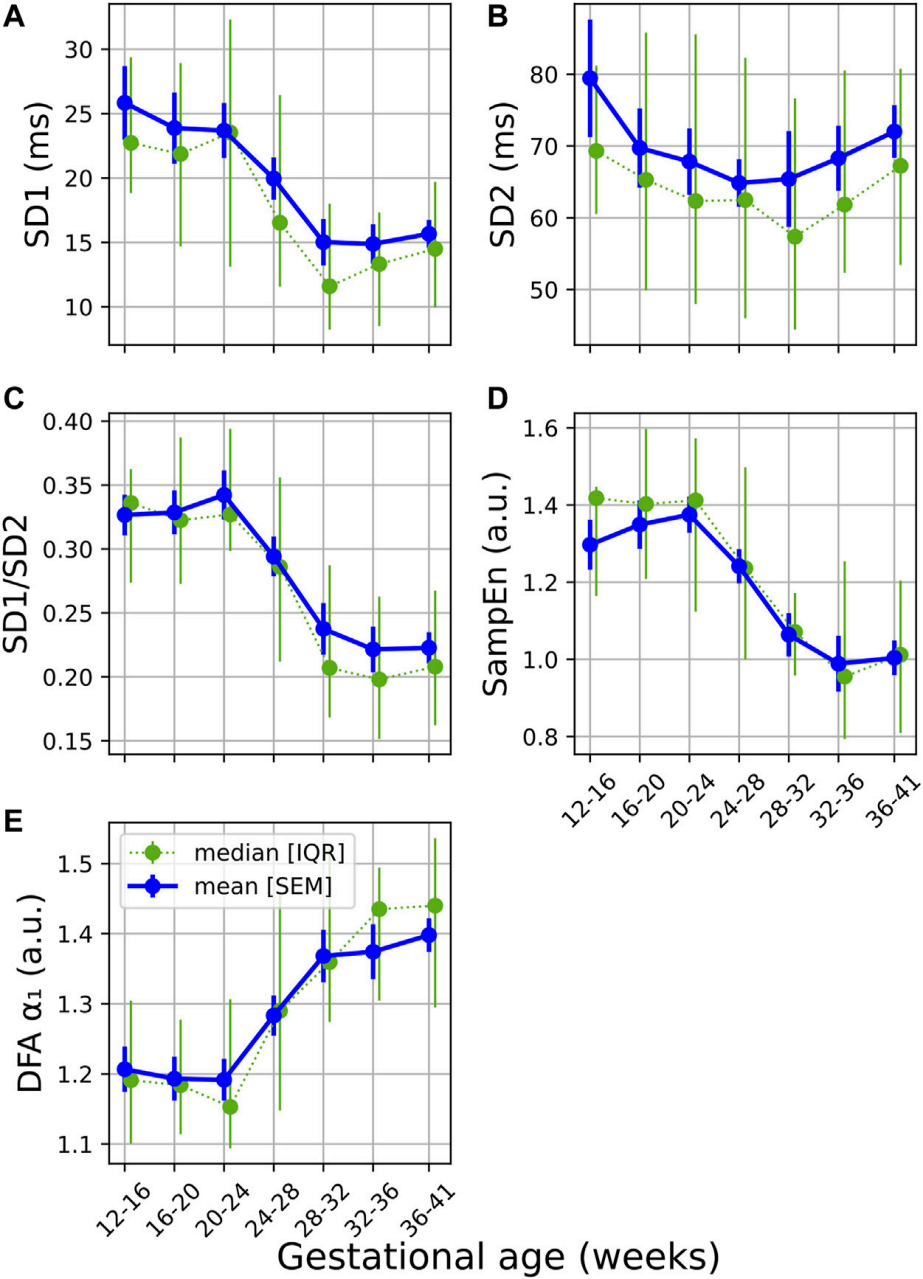
Temporal evolution of non-linear features over GA bins. HRV features for all participants were grouped into bins of 4 weeks. The mean and standard error of the mean (full line) as well as the median and interquartile range (dotted line) of the HRV features per bin is plotted against GA for **(A)** SD1; **(B)**; SD2; **(C)** SD1/SD2; **(D)** SampEn; and **(E)** DFA α₁.

From Figures 2A,C gradual decrease can be seen in both SD1 and SD1/SD2 (calculated from the Poincaré analysis) over GA. Similar to the standard features discussed in the previous section, the sharpest change is seen around 24–32 weeks of gestation. A decrease in this ratio indicates a reduction in short-term variability (typically associated with vagal activity). This is confirmed by the significant changes reported in Table 3.

**TABLE 3:** Results from the grouped analysis for non-linear HRV features.

Features	GA_1_ median (IQR)	GA_2_ median (IQR)	GA_3_ median (IQR)	Friedman *p*-value	GA_1_→GA_2_		GA_2_→GA_3_		GA_1_→GA_3_	
					*p*	*U*_1_	*p*	*U*_1_	*p*	*U*_1_
SD1 (ms)	23.2 (15.4–31.6)	18.0 (11.1–23.4)	14.7 (10.9–18.5)	**<0.0001**	0.101	0.125	1	0	**0.006**	0.125
SD2 (ms)	68.0 (52.9–85.9)	61.6 (46.2–82.9)	65.8 (55.4–81.4)	0.131	1	0.054	1	0.054	1	0.036
SD1/SD2	0.34 (0.27–0.38)	0.26 (0.22–0.35)	0.21 (0.17–0.26)	**<0.0001**	0.093	0.107	0.051	0.089	**<0.0001**	0.196
SampEn (a.u.)	1.40 (1.27–1.53)	1.19 (1.01–1.38)	0.94 (0.83–1.24)	**<0.0001**	**0.050**	0.071	0.061	0.071	**<0.0001**	0.214
DFA α₁ (a.u.)	1.19 (1.07–1.27)	1.29 (1.19–1.44)	1.41 (1.29–1.50)	**<0.0001**	**0.042**	0.107	0.177	0.071	**<0.0001**	0.125

All continuous data are presented as median (IQR). First, the Friedman’s *p*-value is calculated to determine whether significant changes occur over all groups. Thereafter, the Dunn’s post-hoc test with Bonferroni correction is applied to determine the *p*-value between groups. Cohen’s *U*_*1*_ is calculated to determine effect size. Values of approximately 0.15 and 0.33 represent small and medium effects, respectively.

The bold type indicates that they values are statistically significant.

A decrease and increase are seen in SampEn and *α₁*, respectively (Figures 2D,E; Table 3). The decrease in SampEn points to a time series that becomes more regular and predictable. Changes in SampEn are accompanied with an effect size of *U*_*1*_ = 0.214 between GA_1_ and GA_3_, which is the largest effect observed across all HRV features. Increases in *α₁* ranging across 1 and 1.5 indicate stronger correlations within the time series, pointing to a less complex signal. Again, both features display sharp changes over 24–32 weeks of gestation (Figures 2B,C). Note that the changes for all non-linear features are highly significant (*p* < 0.0001, Table 3). Furthermore, the changes from GA_1_→GA_3_ are all highly significant (*p* < 0.0001), while this is not the case for the standard features (Table 2).

### 3.3 Heart Rate Fragmentation

**FIGURE 3:**
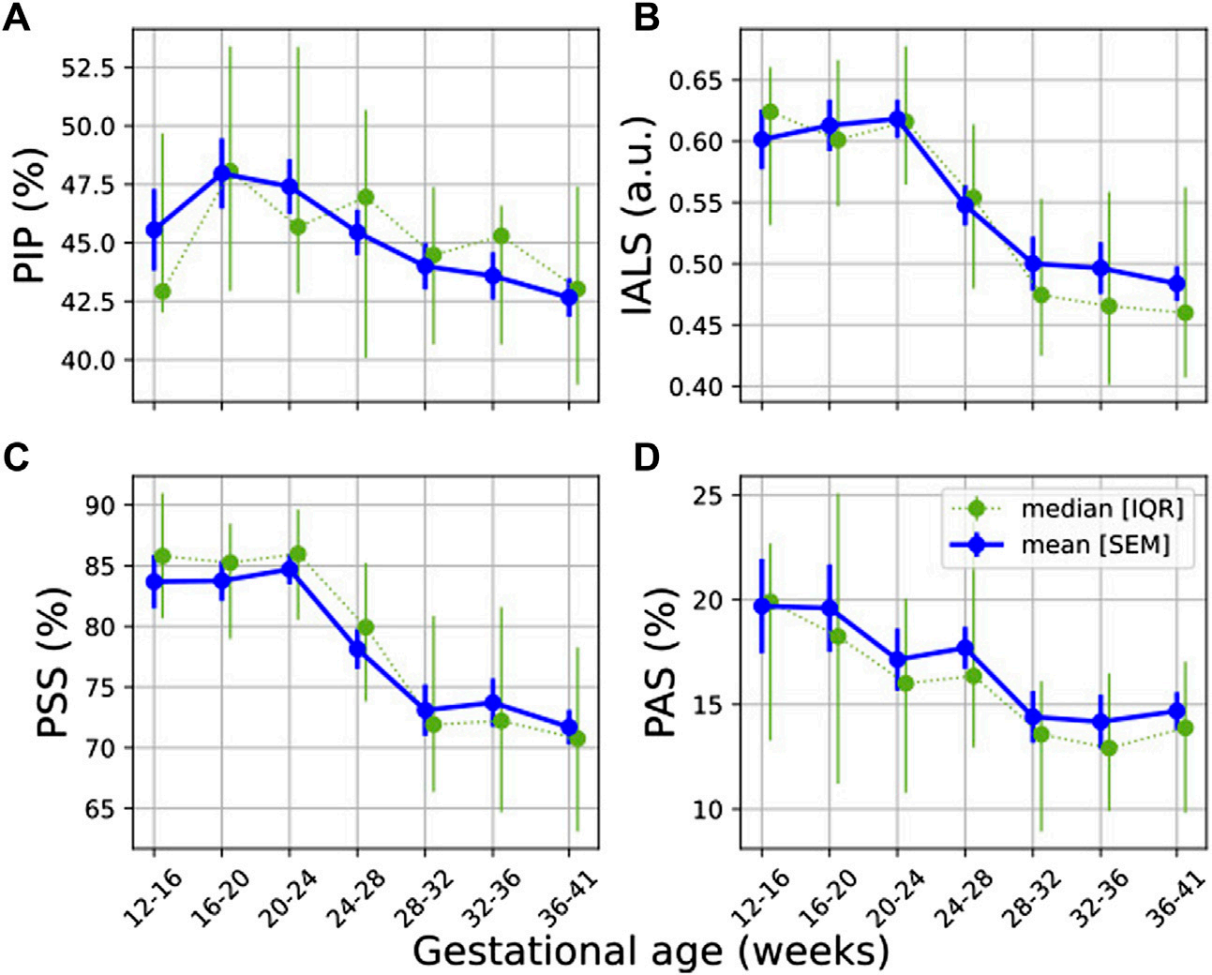
Temporal evolution of HRF features over GA bins. HRV features for all participants were grouped into bins of 4 weeks. The mean and standard error of the mean (full line) as well as the median and interquartile range (dotted line) of the HRV features per bin is plotted against GA: **(A)** PIP; **(B)** IALS; **(C)** PSS; and **(D)** PAS.

Most HRF (IALS, PSS and PAS) showed a downward trend with progressing GA (Figures 3B–D), with IALS, PSS and PAS decreasing significantly (Table 4). Although not significant, PIP does decrease steadily from 20 weeks onward (Figure 3A). All features decrease noticeably between 24 and 32 weeks of GA, although this is particularly evident for IALS and PSS.

**TABLE 4:** Results from the grouped analysis for HRF features.

Features	GA_1_ median (IQR)	GA_2_ median (IQR)	GA_3_ median (IQR)	Friedman *p*-value	GA_1_→GA_2_		GA_2_→GA_3_		GA_1_→GA_3_	
					*p*	*U*_*1*_	*p*	*U*_*1*_	*p*	*U*_*1*_
PIP (%)	47.5 (42.3–52.5)	45.7 (40.4–48.9)	45.4 (40.3–46.5)	0.074	1	0.018	0.909	0.036	0.144	0.196
IALS (a.u.)	0.62 (0.55–0.66)	0.54 (0.48–0.59)	0.49 (0.42–0.55)	**<0.0001**	**0.014**	0.107	0.291	0.054	**<0.0001**	0.179
PSS (%)	86.6 (80.1–88.7)	77.4 (71.8–83.8)	73.4 (65.9–77.8)	**<0.0001**	**0.011**	0.054	0.409	0.018	**<0.0001**	0.089
PAS (%)	17.5 (12.7–20.0)	16.8 (11.8–18.8)	13.9 (11.2–16.2)	**0.031**	1	0.054	0.653	0	0.146	0.071

All continuous data are presented as median (IQR). First, the Friedman’s *p*-value is calculated to determine whether significant changes occur over all groups. Thereafter, the Dunn’s post-hoc test with Bonferroni correction is applied to determine the *p*-value between groups. Cohen’s *U*_*1*_ is calculated to determine effect size. Values of approximately 0.15 and 0.33 represent small and medium effects, respectively.

The bold type indicates that they values are statistically significant.

The largest effect size between GA_1_ and GA_3_ for a significant change occurred for IALS (*U*_*1*_ = 0.179, from Table 4), yet this is still a small effect. While an increase in HRF would have suggested a breakdown in the physiological systems regulating HR, these findings instead suggest that there is a decrease in fragmentation with the increasing demands of pregnancy.

### 3.4 Phase Rectified Signal Averaging

**FIGURE 4:**
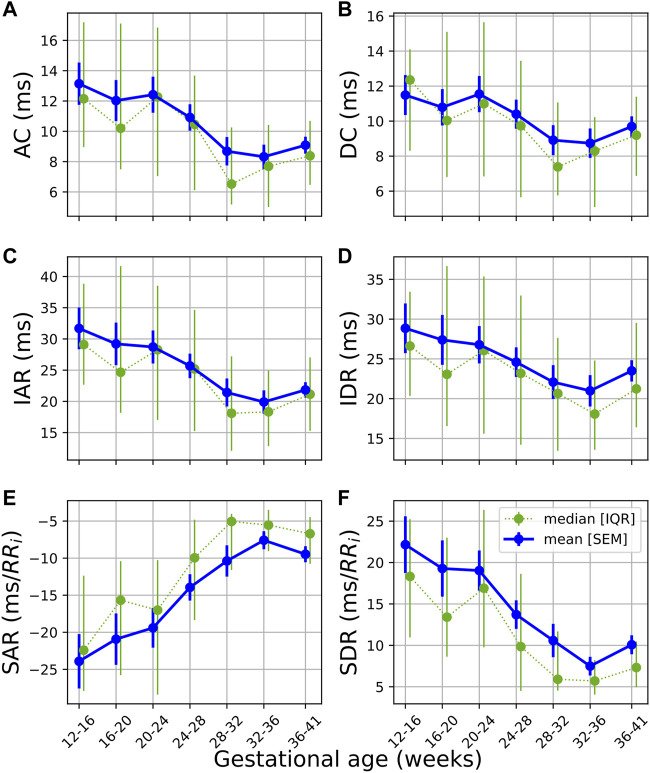
Temporal evolution of PRSA features over GA bins. HRV features for all participants were grouped into bins of 4 weeks. The mean and standard error of the mean (full line) as well as the median and interquartile range (dotted line) of the HRV features per bin is plotted against GA. **(A)** AC; **(B)** DC; **(C)** IAR; **(D)** IDR; **(E)** SAR; and **(F)** SDR.

The temporal evolution of the PRSA features (Figure 4) shows a downward trend across features, with an uptick at the end of pregnancy. Note that SAR is inherently negative but is also decreasing in absolute terms. Also note that AC (4a), IAR (4b), IDR (4e), SAR (4c), and SDR (4f) already start decreasing before 20 weeks GA. (The average responses, i.e., AAR and ADR, displayed no discernable trends and are not shown here).

**FIGURE 5:**
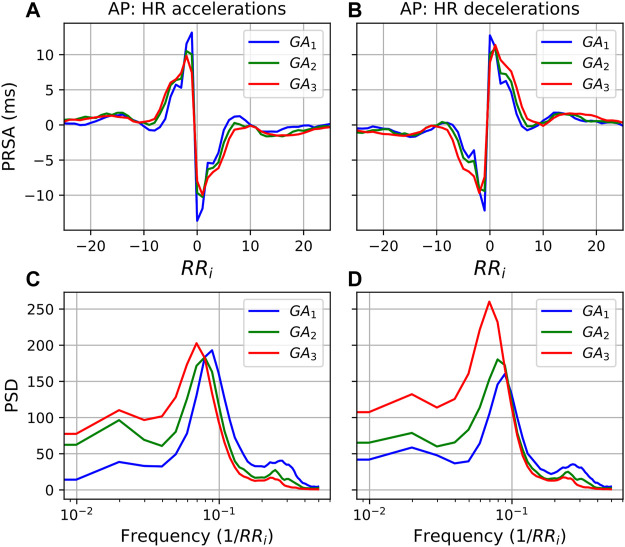
PRSA curves for each GA group with **(A)** accelerations as AP and **(B)** decelerations as AP, and *T* = 1. In all cases, the mean values have been subtracted from the graphs to enable comparison. Furthermore, **(C,D)** show the power spectral densities (PSD) of the PRSA graphs with accelerations and decelerations as AP, respectively.

This dampened response in later gestation, which is also reflected in the PRSA waveforms (Figure 5) and the decreased PRSA features (Table 5), indicates reduced responsiveness in HR. All features except AAR and ADR (the average responses) show significant reductions across GA groups (Table 5). The largest changes are seen in the slopes of the instant responses (SAR and SDR, both with *U*_*1*_ = 0.125 between GA_1_ and GA_3_) and the IAR (U_1_ = 0.143), although their effect sizes are still small.

**TABLE 5:** Results from the grouped analysis for PRSA features.

Features	GA_1_ median (IQR)	GA_2_ median (IQR)	GA_3_ median (IQR)	Friedman *p*-value	GA_1_→GA_2_		GA_2_→GA_3_		GA_1_→GA_3_	
					*p*	*U*_1_	*p*	*U*_1_	*p*	*U*_1_
AC (ms)	12.5 (8.3–17.3)	9.7 (5.9–13.0)	8.2 (6.1–11.2)	**<0.0001**	0.245	0.054	1	0.036	**0.033**	0.107
IAR (ms)	28.9 (19.7–43.2)	24.0 (15.8–30.3)	19.6 (16.5–26.7)	**<0.0001**	0.329	0.089	0.989	0.071	**0.030**	0.143
SAR (ms/RR_i_)	−17.3 (−27.1–−12.3)	−11.7 (−16.0–−6.8)	−7.2 (−10.1–−4.4)	**<0.0001**	**0.032**	0.054	0.348	0.018	**0.0001**	0.125
AAR (ms/RR_i_)	−1.3 (−3.3–0.1)	−2.1 (−2.8–−1.5)	−2.1 (−3.5–−1.4)	0.174	0.207	0.018	1	0.018	0.324	0.036
DC (ms)	11.7 (8.3–14.9)	9.5 (5.8–12.8)	8.4 (6.2–11.7)	**0.001**	0.553	0.054	1	0.018	0.217	0.036
IDR (ms)	27.1 (18.6–39.7)	23.3 (16.2–30.5)	20.8 (16.0–27.9)	**<0.001**	0.678	0.054	1	0.018	0.228	0.071
SDR (ms/RR_i_)	15.8 (11.6–29.3)	11.9 (7.1–16.0)	7.2 (5.6–11.5)	**<0.0001**	**0.047**	0.089	0.444	0.018	**<0.001**	0.125
ADR (ms/RR_i_)	2.6 (1.5–3.6)	2.0 (1.4–2.9)	3.2 (2.2–4.8)	0.091	1	0.018	**0.034**	0.018	0.058	0.089

All continuous data are presented as median (IQR). First, the Friedman’s *p*-value is calculated to determine whether significant changes occur over all groups. Thereafter, the Dunn’s post-hoc test with Bonferroni correction is applied to determine the *p*-value between groups. Cohen’s *U*_*1*_ is calculated to determine effect size. Values of approximately 0.15 and 0.33 represent small and medium effects, respectively.

The bold type indicates that they values are statistically significant.

**FIGURE 6:**
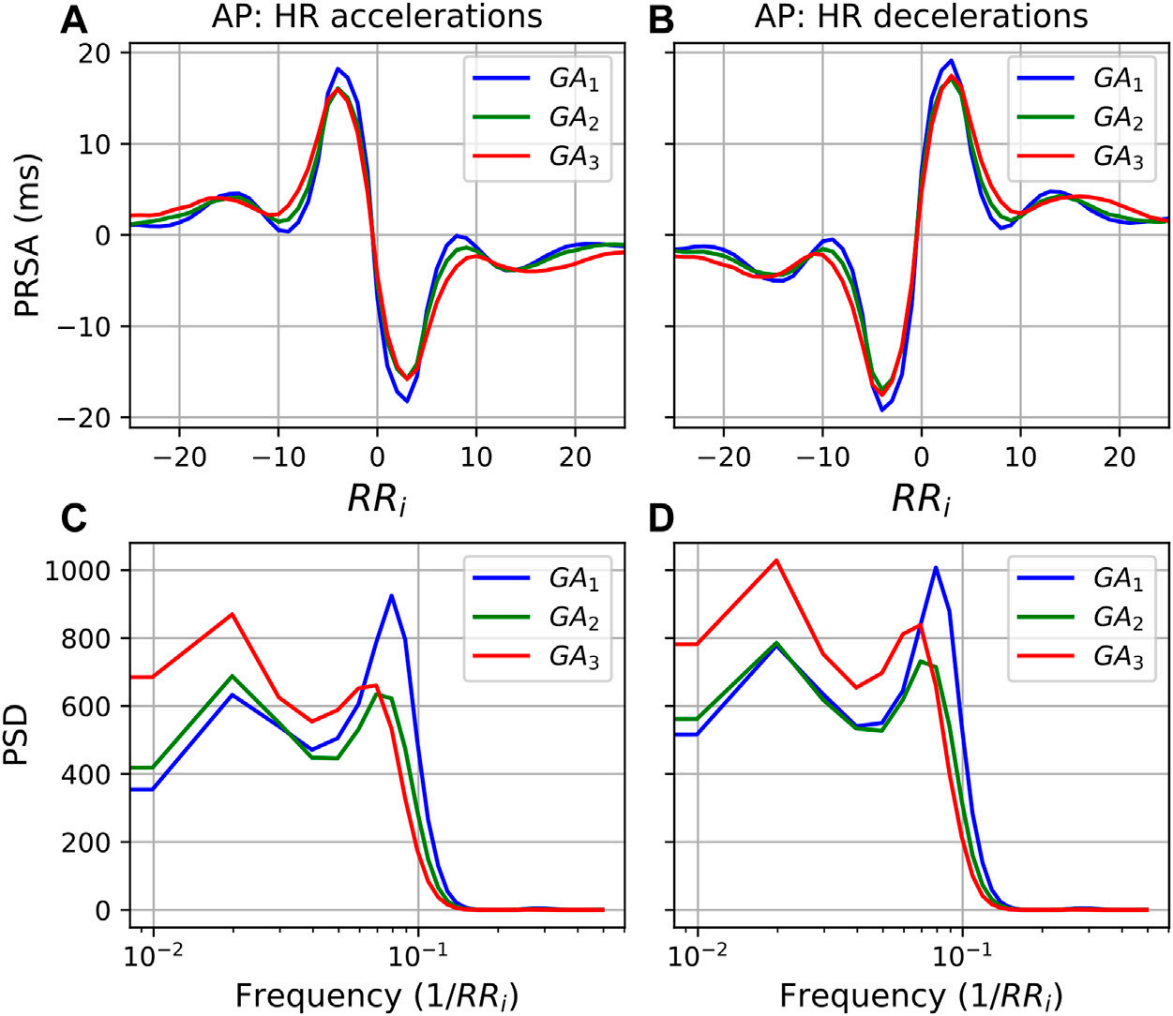
PRSA curves for each GA group with **(A)** accelerations as AP and **(B)** decelerations as AP, and *T* = 5. In all cases, the mean values have been subtracted from the graphs to enable comparison. Furthermore, **(C,D)** show the power spectral densities (PSD) of the PRSA graphs with accelerations and decelerations as AP, respectively.

Moving to the frequency domain, in the PSDs in Figure 6 a similar response is observed for both accelerations and decelerations (*T* = 5). However, for the LF band in Figure 5 (0.035/RR_i_ Hz to 0.15/RR_i_ Hz, translating to approximately 0.04–0.2 Hz) the behavior is markedly different. For accelerations (Figure 5C), activity in the LF band remains similar throughout. However, when decelerations serve as APs, the LF activity increases substantially with progressing pregnancy. (Note that the shifting peaks that can be observed are a result of an increase in mean HR since the frequency is a function of the RR intervals.)

The observation that the LF power in the deceleration response increases relative to that of the acceleration response is also confirmed in Table 6, which reports the ratio between frequency domain values in the acceleration and deceleration response. In fact, although it may not be visually evident from Figure 5, we see that the deceleration values significantly increase relative to the acceleration values for all features. Overall, we also see the largest effect sizes of our analysis. LF_acc_:LF_dec_ and HF_peakacc_:HF_peakdec_ both have *U*_*1*_ > 0.3, while TP_acc_:TP_dec_ has an effect size of 0.607 between GA_1_ and GA_3_. These changes suggest that increasing activity goes into decelerating the HR towards the end of pregnancy.

**TABLE 6:** Results from the grouped analysis for the ratio between PRSA frequency features with accelerations and decelerations as APs, respectively (T = 1).

Features	GA_1_ median (IQR)	GA_2_ median (IQR)	GA_3_ median (IQR)	Friedman p-value	GA_1_→GA_2_		GA_2_→GA_3_		GA_1_→GA_3_	
					*p*	*U*_1_	*p*	*U*_1_	*p*	*U*_1_
LF_acc_:LF_dec_	1.17 (1.05–1.38)	0.98 (0.88–1.09)	0.93 (0.76–0.97)	**<0.0001**	**0.004**	0.089	0.220	0.143	**<0.0001**	0.329
HF_acc_:HF_dec_	1.08 (0.99–1.27)	0.99 (0.89–1.10)	0.93 (0.80–1.02)	**<0.0001**	**0.040**	0.125	0.507	0.018	**<0.001**	0.179
TP_acc_:TP_dec_	1.17 (0.99–1.26)	0.96 (0.86–1.09)	0.83 (0.70–0.94)	**<0.0001**	**0.018**	0.089	**0.015**	0.161	**<0.0001**	0.607
LF peak_acc_:LF peak_dec_	1.16 (0.99–1.42)	0.95 (0.88–1.09)	0.89 (0.70–0.94)	**<0.0001**	**0.024**	0.036	0.142	0.107	**<0.0001**	0.196
HF peak_acc_:HF peak_dec_	1.18 (0.99–1.42)	0.93 (0.86–1.14)	0.82 (0.62–0.94)	**<0.001**	0.071	0.071	**0.039**	0.125	**<0.0001**	0.375

All continuous data are presented as median (IQR). First, the Friedman’s *p*-value is calculated to determine whether significant changes occur over all groups. Thereafter, the Dunn’s post-hoc test with Bonferroni correction is applied to determine the *p*-value between groups. Cohen’s *U*_*1*_ is calculated to determine effect size. Values of approximately 0.15 and 0.33 represent small and medium effects, respectively.

The bold type indicates that they values are statistically significant.

Lastly, Table 7 lists the number of accelerations, decelerations, and constant points (i.e., with no change from one RR to the next) that were detected for *T* = 1 and *T* = 5, respectively. In both cases the number of constant points remains relatively stable, while decelerations decrease relative to accelerations. This shift is more pronounced for *T* = 1.

**TABLE 7:** The number of accelerations, decelerations, and constant points (i.e., no change from one RR to the next) for *T* = 1 and *T* = 5, respectively.

	*T* = 1				*T* = 5			
	Accelerations	Decelerations	Constants	Ratio	Accelerations	Decelerations	Constants	Ratio
GA1	102,829	105,248	19,734	1:1.02:0.19	113,144	108,705	5402	1:0.96:0.05
GA2	127,584	123,119	21,074	1:0.97:0.17	136,696	128,779	5678	1:0.94:0.04
GA3	138,837	127,466	21,029	1:0.92:0.15	147,070	134,498	5108	1:0.91:0.03

Ratio refers to the ratio of accelerations, decelerations, and constants.

## 4 Discussion

In this paper, we comprehensively analyzed HRV at a high temporal granularity to track the dynamic progression of maternal autonomic modulation over normal pregnancy. We generated a holistic overview of autonomic changes by incorporating non-linear, HRF, and PRSA features to the standard time and frequency domain analysis. These features have rarely or, in the case of HRF, never been assessed in a pregnant population. We found that some of these features are more sensitive to GA than standard time and frequency features. Furthermore, contrary to previous research in this field, we also calculated the effect sizes of the changes in mHRV. Overall, our findings indicate that decreased vagal modulation, dampened autonomic response, reduced complexity and decreased HR fragmentation accompany advancing gestation. Our results show that even though changes in HRV features are often statistically significant, their effect sizes are small—indicating that the increasing physiological demands of progressing pregnancy are well tolerated by the maternal ANS. Interestingly, while overall autonomic activity remained fairly stable, we found a burst of autonomic activity occurring between approximately 24 and 32 weeks of gestation.

This burst of autonomic activity, which coincides approximately with the transition from the second to the third trimester, is reflected in the temporal analyses of almost all HRV features (Figures 1–4). To our knowledge, this change has not been reported in literature and demonstrates the value of assessing autonomic modulation at regular intervals throughout pregnancy. Physiological changes during gestation are non-linear for both mother and fetus (Tan and Tan, 2013; Tan and Lewandowski, 2020). Therefore, it seems unlikely that changes in autonomic modulation would be linear. We speculate that this burst in maternal autonomic activity could be attributed to fetal autonomic modulation. The transition from late-second to early third-trimester is associated with an acceleration in fetal autonomic maturation (Schneider et al., 2018). Since autonomic modulation is mirrored in cardiovascular regulation and there is evidence for maternal-fetal cardiac coupling (Khandoker et al., 2016; Avci et al., 2018), it may be that the maternal autonomic activity is reflecting that of the fetus.

Current literature on maternal autonomic modulation typically investigates the interplay of the autonomic branches by assessing standard HRV features from both time and frequency domain. Concurrent with most research, we find that vagal modulation reduces as reflected in pNN50, RMSSD and HF (Table 2 and Figure 1). An upward trend was also noted in LF/HF, indicating a shift in sympathovagal ratio, aligning with this decrease in vagal activity. Additionally, even though mean HR increased over pregnancy as expected (Green et al., 2020), SDNN—a measure of overall variability – decreased until about 32 weeks of gestation before increasing again. This may suggest that while vagal activity decreases, compensatory processes stabilize the overall variation in HR in the third trimester.

Our findings that parasympathetic activity reduces towards the end of gestation are in agreement with the results of some investigators (Balajewicz-Nowak et al., 2016; Kolovetsiou-Kreiner et al., 2018; Garg et al., 2020), but contrast the work of others who found that no significant autonomic changes occur across gestation (Voss et al., 2000; Moertl et al., 2009). It should be noted that in the studies contrasting ours, one only compares between two GA groups (Voss et al., 2000) and the other focused on early pregnancy (Moertl et al., 2009). Concerning sympathetic activity, we found a decrease in LF (Table 2 and Figure 1). This contradicts the findings of most researchers that there is an increase (Kolovetsiou-Kreiner et al., 2018; Garg et al., 2020) or no significant changes in LF during pregnancy (Voss et al., 2000; Balajewicz-Nowak et al., 2016). It should be noted that the validity of LF as a measure of sympathetic activity is often disputed (Heathers, 2014). However, findings from microneurography studies—which more directly assess sympathetic activity—indicate that there is increased sympathetic activity in pregnancy accompanied by decreased sympathetic signaling to end-organs, such as the heart (Usselman et al., 2015; Steinback et al., 2019). Subsequently, the decrease in LF may be a result of reduced sympathetic influence on HR and decreased parasympathetic activity, which overlaps with the sympathetic activity in the LF band.

Poincaré plots and associated features are commonly used in HRV analyses (Khandoker et al., 2013), yet (to our knowledge) this method has not been calculated longitudinally over normal pregnancy. We found that the ratio obtained from this plot (SD1/SD2) is sensitive to progressing pregnancy (Table 3 and Figure 2C). The decrease is mainly driven by a decrease in SD1 which signals reduced vagal activity, which aligns with the results of pNN50, RMSSD and HF (Figure 1).

Furthermore, our research indicates that HR complexity decreases throughout normal pregnancy as confirmed by both SampEn and *α*_*1*_ (Table 3 and Figures 2D,E). While one study found increasing complexity with progressing pregnancy (Peña et al., 2011), others confirm our results that complexity decreases towards the end of gestation (Baumert et al., 2012). Furthermore, complexity was found reduced in pregnant women compared to non-pregnant controls (Yeh et al., 2009), likely due to the increased demands on the maternal cardiac system during pregnancy. It is also noticeable that changes in complexity measures seemed more sensitive to advancing gestation than most standard HRV features, with SampEn having a larger effect size than standard features (*U*_*1*_ = 0.214, Table 3).

Since pregnancy is essentially an alteration in a normal physiological system, complexity measures may indicate how well the body is responding to this change. Subsequently, reducing complexity could indicate that it becomes increasingly difficult for the maternal ANS to quickly respond under the increasing demands of gestation. Indeed, this hypothesis is also supported by other findings. Note that for most features—pNN50 and RMSSD in Figure 1 are good examples—which decrease with advancing gestation, its standard error of the mean also becomes narrower (Figure 1D), indicating that large variations in beat-to-beat HR are uncommon for most participants by the end of pregnancy. Additionally, the PRSA analysis (Figures 4–6) showed a dampened response towards the end of gestation. This is further echoed in the significant reduction in almost all PRSA features (Table 5). However, the accompanying effect sizes are small and the dampening in HR responsiveness is not comparable to that seen in diseased states (Bauer et al., 2006b). Furthermore, the two other studies that have calculated PRSA to study maternal HRV found no significant correlations with GA (Casati et al., 2016; Carpenter), although only DC was studied and assessing its correlation with GA was not the primary aim of these studies.

This dampened autonomic response does not necessarily indicate a deterioration in autonomic function. On the contrary, in our participant group, HRF indices decrease during healthy pregnancy (Table 4 and Figure 3). While an increase in HRF would suggest a breakdown in the hierarchy of physiological systems controlling HR, these findings suggest that the integration of systems controlling HR does not fragment with the increasing physiological demands of pregnancy. Rather, it seems that a smoother control of HR is exhibited in later pregnancy. This could suggest that the dampened autonomic response is not a sign of strain, but rather indicative of a more stable autonomic system that is tightly regulated to balance the complex demands of pregnancy. However, the trade-off for this stability might be that the mother is unable to optimally respond to environmental perturbations such as stress in late pregnancy.

The exact mechanisms that promote stable ANS activity in this situation are not known. However, the PSD of the PRSA graphs (Figures 5C,D, 6C,D) offer some insight into how processes mediating HR accelerations and decelerations change with progressing gestation. In the low frequency range (0.035/RR_i_ Hz to 0.15/RR_i_ Hz, i.e., approximately 0.04–0.2 Hz), similar behavior is seen when *T* = 5 (Figure 6). However, for *T* = 1, while the frequency response for accelerations (Figure 5C) is stable across GA groups, there is a remarkable increase with gestation for decelerations. This is also reflected in the significant decrease in the ratio (acceleration: deceleration) of all PRSA frequency parameters (Table 6), in particular for TP which has an accompanying effect size of *U*_*1*_>0.6, by far the largest of our analysis. Since pregnancy results in an increase in basal HR (Green et al., 2020), we hypothesize that there is an increase in activity in this LF frequency region (which is associated with both branches of the ANS) of decelerations to ensure that the increasing mean HR stays within a healthy range. This would align with what is seen in Figure 1A, where the increase in mean HR starts to plateau after 32 weeks GA. Additionally, when studying the normal ranges for mean HR throughout the pregnancies of over 1000 women, Green et al. (2020) found not only a similar plateau, but also a slight decrease in mean HR during the final weeks of pregnancy.

Furthermore, Figures 5C,D show the diminishing effect of respiratory modulation in the HF band over time. As the abdomen grows with advancing gestation, the depth of breathing reduces. Subsequently, as can be explained through the Frank-Starling, and lower HR modulation ensues. This is reflected in the decreases in pNN50 and RMSSD (Figures 1C,D), which have also been observed by others (Balajewicz-Nowak et al., 2016; Garg et al., 2020).

Overall, while the changes in mHRV are significant, the effect sizes of these changes are typically small. This stable autonomic modulation offers good news for obstetric screening opportunities as it suggests that there is a stable autonomic baseline to track gestational changes against. As pregnancy complications such as preeclampsia are associated with insufficient autonomic regulation, detecting changes in HRV features with larger effects than we observed in this study may facilitate better screening for such complications. Furthermore, PRSA features such as AC, IDR, IAR, SDR and SAR show decreasing trends earlier than all other features and, importantly, before 20 weeks of gestation. Currently, hypertensive disorders of pregnancy can only be diagnosed after 20 weeks of gestation. Subsequently, investigating these features in populations who develop pregnancy complications may contribute to the eventual early detection of such complications. Additionally, an uptick can be seen in PRSA features (Figure 4) just before the end of pregnancy. If this sudden change is associated with the body preparing for delivery, it could be of value to investigate whether such findings are also observed in cases of preterm delivery.

It should be noted that autonomic activity is not the only driving force behind the physiological changes in pregnancy. Major hormonal changes occur throughout pregnancy. Yet, their link to autonomic activity (as assessed by HRV) remains unclear (von Holzen et al., 2016), likely in part due to the difficulty of isolating the effect of a hormone in an already complex physiological system. Some researchers conclude that estrogen is linked to increased parasympathetic activity (Dart, 2002; Gökçe et al., 2005), while others found a negative relationship between progesterone and vagal activity (von Holzen et al., 2016; Schmalenberger et al., 2020). A combination of estrogen and progesterone (as is the case in pregnancy) seems to have no effect on HRV (Fernandes et al., 2005; Gökçe et al., 2005), though it should be noted that these studies were not performed in pregnant populations.

Finally, we note that while this study offers novel information on gestational autonomic modulation, the results are limited by the modest sample size. Similar assessments are necessary in larger populations to confidently draw conclusions about pregnant populations. Although our dataset does have a uniquely high median number of measurements per participant, some participants naturally have less than eight. Moreover, the timings of participants’ measurements relative to their GA do vary when compared to the protocol. Subsequently, it was necessary to divide the data into three GA groups to facilitate appropriate statistical testing, taking the average per participant if they have multiple measurements in a group. Additionally, the dataset is further limited regarding participant information for the mother, in part because the focus of the original analysis was on fetal HRV. Subsequently, information on factors that may influence mHRV during recording sessions (e.g., fasting, coffee consumption, and smoking habits) are unavailable and cannot be accounted for in our analysis.

Furthermore, during the preprocessing for frequency domain and some complexity features, unreliable RR intervals were interpolated. This interpolation is necessary for determining these HRV features, but may affect their results and subsequent interpretation, particularly regarding HF (Peters et al., 2008). However, since the changes in HF in our analysis are reflect those of pNN50 and RMSSD as is expected from literature (Shaffer and Ginsberg, 2017), we believe the trend observed in HF is reliable. Still, frequency domain features should be interpreted with caution, since their calculation relies on an assumption of stationarity which is not guaranteed and involves averaging multiple segments which may represent different physiological states. Lastly, our measurements start at 14 weeks of gestation, while major cardiovascular and autonomic changes are also known to occur within the first few weeks of pregnancy. Ideally, future work would incorporate measurements from as early in gestation as possible.

In conclusion, this work offers a comprehensive look at autonomic modulation in normally progressing pregnancy. By assessing HRV at a high temporal granularity with a variety of features, we find that although significant reductions in vagal activity, complexity, HR responsiveness, and HRF do occur, these changes are of small effect. Therefore, in a healthy pregnancy, the increasing stress of advancing gestation is tolerated well.

## Data Availability

The data analyzed in this study is subject to the following licenses/restrictions: The data in question is sensitive and owing to privacy restrictions, cannot be made publicly available. Requests to access these datasets should be directed to m.bester@tue.nl.
